# Supplementary material for: Curcumin Inhibits TORC1 and Prolongs the Lifespan of Cells with Mitochondrial Dysfunction
Source: Cells. 2024 Sep 1;13(17):1470. doi: 10.3390/cells13171470 (PMC11394456; doi:10.3390/cells13171470)
Supplement: Supplementary file 1 [file cells-13-01470-s001.zip › cells-3138141-supplementary.pdf]

Figure 3B

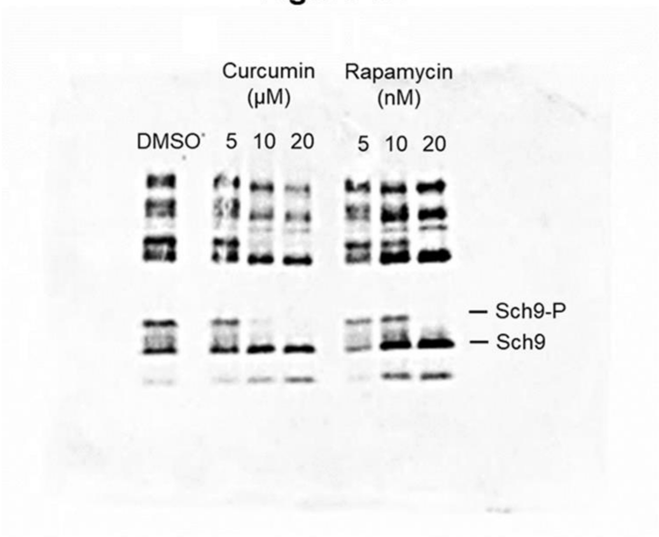

Figure 3D

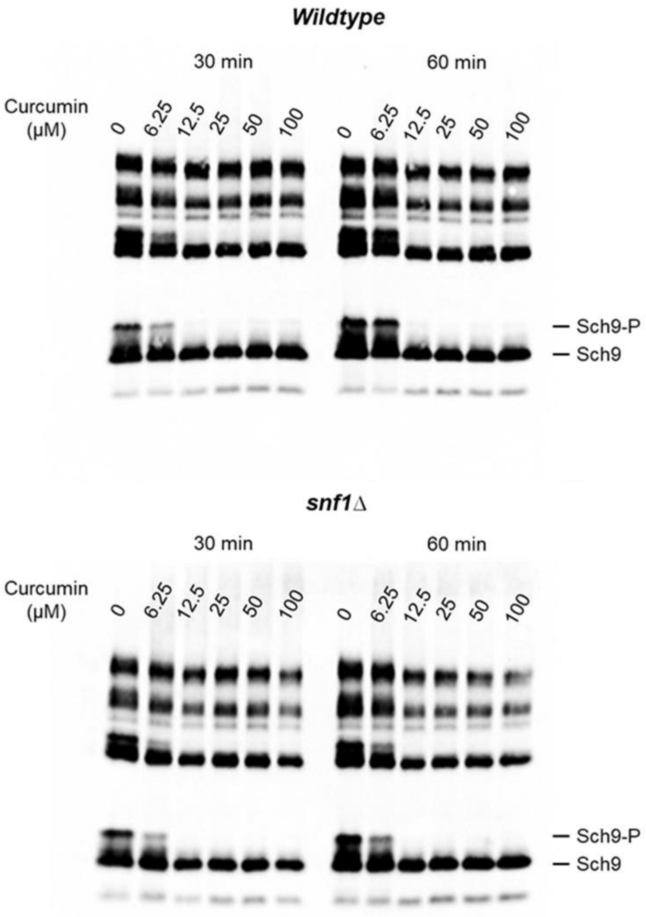

Figure S1. Western blots of figures 3B and 3D. The original, uncropped, and unadjusted images of Western blots illustrate the assessment of TORC1 activity. This was achieved by monitoring the phosphorylation of the Sch9 substrate through Western blotting."

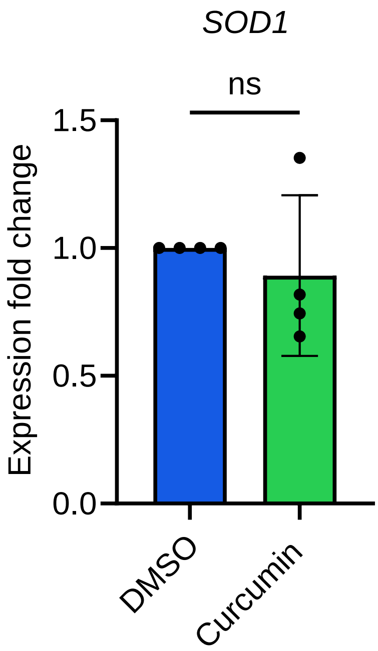

Figure S2. Effect of curcumin on expression of and *SOD1* gene. Expression analysis of *SOD1* gene was analysed by qRT-PCR of yeast cells treated with DMSO and curcumin (10 μM). Gene expression of curcumin treated samples were compared with DMSO control. Data are represented as means ± SD (n=4). ns (non-significant) based on based on two-sided Student’s t-tests.
